# A role for SUV39H1-mediated H3K9 trimethylation in the control of genome stability and senescence in WI38 human diploid lung fibroblasts

**DOI:** 10.18632/aging.100678

**Published:** 2014-07-21

**Authors:** Corinne Sidler, Rafal Woycicki, Dongping Li, Bo Wang, Igor Kovalchuk, Olga Kovalchuk

**Affiliations:** Department of Biological Sciences, University of Lethbridge, Lethbridge, AB, T1K 3M4, Canada

**Keywords:** senescence, gene expression, genome stability, H3K9 methylation, SUV39H1a

## Abstract

Cellular senescence has been associated with the age-dependent decline in tissue repair and regeneration, the increasing deterioration of the immune system, and the age-dependent increase in the incidence of cancer. Here, we show that senescence of human lung fibroblast WI-38 cells is associated with extensive changes to the gene expression profile, including the differential expression of transcriptional and epigenetic regulators. Among those, *SUV39H1* was downregulated in senescent cells, correlated with a decrease in global H3K9 trimethylation, reduced H3K9me3 levels in repetitive DNA sequence regions such as satellites and transposable elements, and increased transcription of these repetitive DNA sequences. This indicates that *SUV39H1* plays a role in limiting genomic instability in dividing cells and suggests that *SUV39H1* downregulation may contribute to the establishment of senescence by increasing genomic instability. Additionally, the manipulation of SUV39H1 expression levels resulted in altered cell cycle distribution, suggesting a causal role of *SUV39H1* in the establishment of cellular senescence. Thus, based on our findings and the results from previous reports, we propose a model in which *SUV39H1* downregulation promotes the establishment of cellular senescence.

## INTRODUCTION

Cellular senescence plays a multifaceted role in human aging and age-related pathologies; senescent cells exhibit altered secretory profiles, which may facilitate cell transformation and cancer progression [1]. The senescence of immune cells has been associated with the deterioration of the immune system [2], resulting in an increased susceptibility to infectious diseases and a reduced capacity to mount immune responses toward new antigens [3] such as those associated with cancer cells or vaccinations [4]. The senescence of stem cells limits the repair and regenerative capacity of aging tissues and organs and thus promotes age-associated functional decline [5]. On the other hand, the deregulation of senescence-associated genes is sufficient to induce the malignant transformation of human diploid cells [6]. Thus, a molecular understanding of the underlying mechanisms of senescence allows the development of strategies to prolong the human healthy lifespan and may point towards new potential drug targets for the treatment of cancer.

The study of senescence often employs human diploid fibroblasts, which exhibit a limited in vitro replicative lifespan of approximately 50 ± 10 population doublings (PD) in the case of lung fibroblasts, after which the cell cultures progressively decline [7, 8]. Senescent cells have been shown, in vivo, to accumulate in tissues with increasing age [9] and the in vitro lifespan of primary cell cultures has been correlated with the age and maximum lifespan of the donor species [10, 11]; thus, human diploid fibroblasts have become a widely used model for age-related molecular and physiological changes.

There are numerous theories on what may be the driving cause of senescence, namely telomere shortening [12] resulting in the exposure of the chromosome ends and in the permanent activation of a DNA damage signal leading to senescence [13-15]; changes in DNA repair efficiency and fidelity that result in the accumulation of unrepaired DNA damage and, thus, in DNA damage signalling-induced senescence [16]; and oxidative stress associated with an increase in oxidative damage to DNA and other macromolecules of the cell [17]. While these different types of cellular stresses are well accepted for their roles in the establishment of senescence, the regulatory pathways that mediate it are not as well understood.

Several pathways have been implicated in the induction of senescence, including mTOR [18], p53 activation [15] linking the DNA damage response to the G1 cell cycle arrest, the transcriptional repression of E2F target genes through RB/E2F-directed targeting of heterochromatin formation [19, 20], and the p38 MAPK-mediated increase in Lamin B1 expression resulting in changes to the nuclear shape and in senescence in response to oxidative stress [21], among others. Senescence is currently viewed as a genetic and well as epigenetic phenomenon [22], and changes in chromatin structure have been increasingly considered for their function in aging and senescence (reviewed in [23-25]). The decreased expression of histone genes and the reduction of heterochromatin marks, such as DNA methylation, and repressive histone marks, such as H3K9me3, H3K27me3, and H4K20me3, during senescence and aging suggest that aging is associated with the loss of heterochromatin. This may ultimately result in changes in gene expression and genome integrity.

Here, we show that senescence in WI-38 human diploid lung fibroblasts is associated with extensive changes to the gene expression profile, including downregulation of transcriptional or epigenetic regulators. This included the reduced expression of *SUV39H1* and corresponding reduction in H3K9 trimethylation. Both the overexpression of *SUV39H1* and the inhibition of SUV39H1 interfered with the cell cycle distribution, suggesting a role of SUV39H1 in the control of senescence.

## RESULTS

### Setup of the senescence model system WI-38

WI-38 human foetal lung fibroblasts have a limited in vitro lifespan of approximately 50 ± 10 PD [7, 8]. A cell stock obtained from ATCC at 19 PD was subcultured to reach three different passages (P10, P15, and P24). In order to determine the senescence status of those cells, we monitored their PD levels, their senescence ratios as determined by a flow cytometry-based SA-β-GAL assay, and their cell cycle distribution, as senescence is characterized by the permanent cell cycle arrest in the G1/G0 phase of the cell cycle as determined by the DNA amount per cell [26].

Along with the increasing PD level of the cell cultures, the P15 and P24 cultures contained a significantly higher ratio of senescent cells than the P10 cultures (Table 1). On the other hand, the cell cycle analysis revealed an initial increase in the number of cells in the G0/G1 phase along with a decrease in cells in the S or G2/M phases of the cell cycle, when comparing P15 to P10 cells (Table 1). When comparing P24 cells to P10 or P15 cells, a further decrease in S phase cells was observed, paralleled by a decrease in G1/G0 and an increase in G2/M cells (Table 1).

**Table 1:** Senescence properties of cell cultures Population doubling (PD). G0/G1, S, and G2/M columns show the average percentage of cells in the given phase of the cell cycle ± standard deviation (SD). SA-β-Gal pos (%) shows the average percentage of SA-β-Gal positive cells ± SD.

Passage	PD	G0/G1	S	G2/M	SA-β-Gal pos (%)
**P10**	38.3 ± 0.6	73.6 ± 0.4	5.8 ± 0.7	20.7 ± 0.7	56.4 ± 7.1
**P15**	46.8 ± 0.3 *	77.6 ± 1.1 *	4.1 ± 0.4 *	18.4 ± 0.7 *	72.7 ± 4.5 *
**P24**	54.2 ± 0.1 *	71.9 ± 0.2 *	3.1 ± 0.1 *	25 ± 0.3 *	74.5 ± 1.8 *

However, as the determination of the cell cycle profile relies on the detection of the amount of DNA – half the amount of DNA in G1 when compared to G2 phase – it does not allow a distinction between tetraploid cells and G2 cells. Such an accumulation of large cells with altered ploidy levels as cultures became senescent has been described [26], and therefore the most accurate measure to compare the level of cell division in the different cell cultures may be given by the comparison of the number of cells in the S phase at each PD level.

Based on the significant increase in the senescence ratio of the P15 and P24 cultures along with the reduced ratio of cells in the S phase, these three cultures were chosen for all further experiments and will be referred to by their PD numbers (P10 as PD 38, P15 as PD 47, and P24 as PD 54).

### Senescence-related changes to gene expression profiles are accompanied by changes in expression of transcriptional and epigenetic regulators

**Figure 1:**
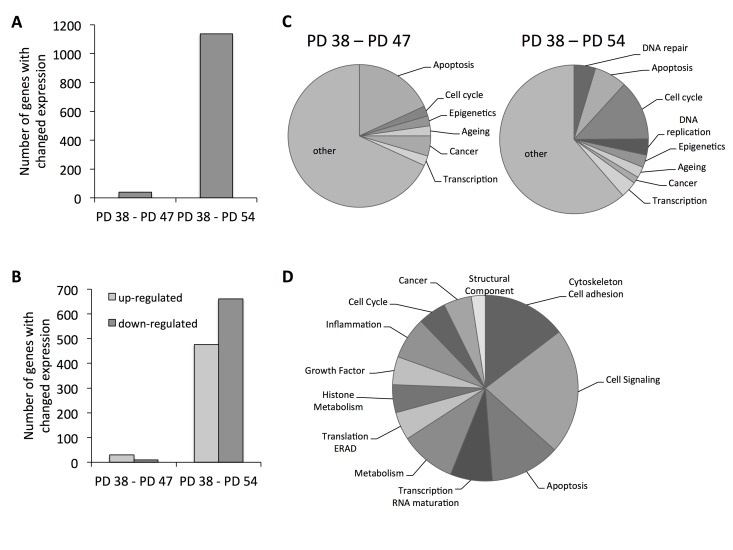
Functional classification of the gene expression results (**A**) Total number of genes affected by expression changes when comparing PD 47 or PD 54 to PD 38 cells. (**B**) Number of genes with changed expression that were up- (light grey) or down-regulated (dark grey). (**C**) Functional classification of differentially expressed genes. (**D**) Functional classification of genes that were differentially expressed between PD 38 and PD 47 and between PD 38 and PD 54 cultures.

To obtain an understanding of what molecular pathways may be involved in the establishment of senescence or may be regulated during senescence, the gene expression profiles of the three different cultures (PD 38, PD 47, and PD 54) were determined using Illumina^®^ Gene Expression BeadChips (S1). Forty genes were differentially expressed when comparing PD 38 and PD 47 cultures, whereas 1,137 genes were differentially expressed between PD 38 and PD 54 (Fig. 1A). Of those genes, the majority were upregulated when comparing PD 47 to PD 38, whereas the majority were downregulated when comparing PD 54 to PD 38 (Fig. 1B). To understand the functional implications that these gene expression changes may have, they were classified by function (Fig. 1C, S2). Gene expression changes that occurred between PD 38 and PD 47 mainly affected apoptosis, but also transcriptional and epigenetic regulation. Changes that occurred between PD 38 and PD 54 mainly affected cell cycle regulation, but also DNA repair, apoptosis, and transcriptional and epigenetic regulation. Further, 80% of the genes that were differentially regulated between PD 38 and PD 47 were also differentially regulated between PD 38 and PD 54, which may indicate that these genes are involved early in the establishment of senescence and mainly play roles in cell signalling, apoptosis, and the modulation of the cytoskeleton and extra-cellular matrix (Fig. 1D). However, a few of the commonly deregulated genes are also involved in transcriptional or epigenetic regulation. This may indicate that changes in cellular signal transduction, as well as changes in transcriptional and epigenetic regulation, may be involved during the onset of cellular senescence.

In addition to the cell cycle arrest of senescent cells in the G0/G1 phase [26], changes in gene expression patterns have been observed in various aging model systems [27]. Therefore, a differential expression of cell cycle regulators as well as senescence-associated genes would be expected in the cultures with higher PD levels. In order to test this, genes that were functionally classified to the cell cycle or aging categories were analyzed in more detail.

This showed differential expression of increasing fractions of genes involved in cell cycle regulation with increasing PD level (Fig. 1C,D); these genes were involved in the regulation of all stages of the cell cycle (S3). The largest group of genes was involved in the regulation of the progression of mitosis and these genes were mostly downregulated, indicating that mitosis occurred at a lower frequency in PD 54 than in PD 38 cultures. Further, most of the differentially expressed genes involved in DNA replication and all of the genes involved in regulation of S phase progression were downregulated, suggesting that DNA synthesis occurred at a lower frequency in the older cultures. Taken together, the observed gene expression changes indicate that high numbers of cells in PD 54 when compared to PD 38 are either in the G1/G0 or G2 phase of the cell cycle, supporting the cell cycle profile analysis (Table 1).

**Figure 2:**
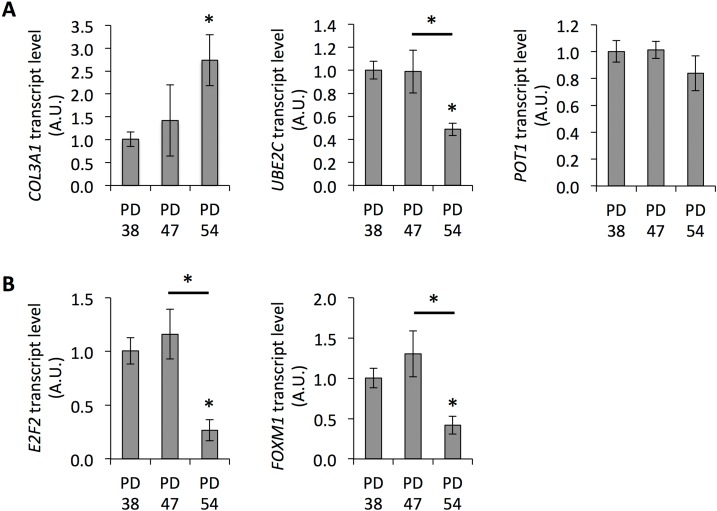
Senescence-associated gene expression Transcript levels of *COL3A1*, *POT1*, and *UBE2C* (**A**), and *E2F2* and *FOXM1* (**B**). Averages of three biological and two technical replicates ± error progression of the SDs of the technical repeats.

Further, age-related gene expression changes were observed in both PD 47 and PD 54 cultures compared to PD 38 cultures (Fig. 1C). These gene expression changes were compared to microarray information from the Human Ageing Genomic Resources and to the predicted functions of genes in aging or senescence (S4). Eleven out of the 16 genes presented were affected by expression changes corresponding to previously described age-related changes in expression [27]. The expression levels of several of these genes were confirmed by qRT-PCR (Fig. 2A,B).

In summary, among the genes that were differentially expressed in PD 54 cultures were numerous cell cycle regulators as well as genes with previously determined age-related expression changes. More detailed functional analysis of the cell cycle regulators and age-related gene expression changes supports the observation that cultures with higher PD levels contain higher numbers of senescent cells (Table 1). Thus, the subsequent step involved the investigation of the changes in the expression of transcriptional and epigenetic regulators in order to determine potential regulatory mechanisms underlying the senescence-associated changes in gene expression profiles.

### A potential role of transcription factors in the establishment of senescence

The gene expression changes between PD 38 and PD 54 were extensive and included numerous transcription factors; further, a large regulatory network surrounding E2F1 could be detected in the dataset. E2F1 is a transcriptional activator involved in the regulation of G1/S progression and DNA replication [28]. Further, E2F1, E2F2, and E2F3 have been shown to interact with retinoblastoma (RB) protein during cell differentiation, resulting in the silencing of E2F target genes [29]. E2F1, E2F2 and E2F3 have been shown to modulate the expression of E2F target genes [28], but also play distinct roles [30]. Therefore, the observed transcriptional repression of *E2F2* in the senescent cultures (Fig. 2B) may induce a shift in the regulation of E2F target genes and, at least in part, explain the differential expression of the genes in the regulatory network of E2F1.

Thus, it can be hypothesized that the deregulated transcription factors may play a role in modulating the expression profiles in senescing cells. To test this, the promoter regions of the genes that were differentially expressed were searched for TFBS recognized by the differentially expressed transcription factors. For this, only transcription factors with binding sites that had less than 1,000 possible variants of their cognate binding motif were considered. Transcription factors were ranked according to how many potential target genes their expression levels correlated with and the overall enrichment of their TFBS within promoters of differentially expressed genes when compared to all promoters was calculated. None of the TFBS of the differentially expressed transcription factors were overrepresented within the promoters of the differentially expressed genes (data not shown).

### Senescence correlates with reduced SUV39H1 expression and H3K9 trimethylation

**Figure 3:**
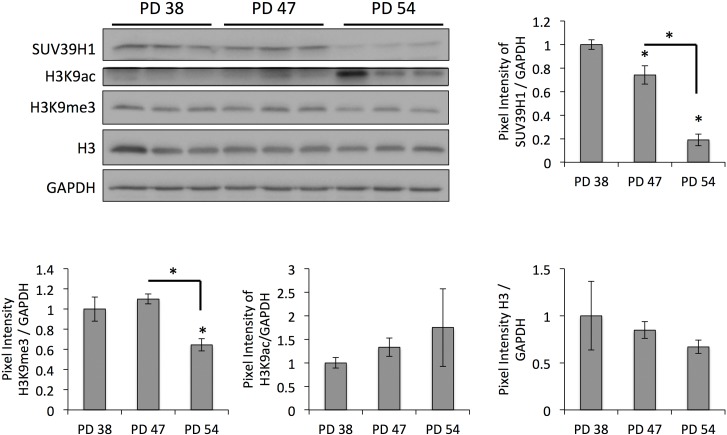
Age-dependent downregulation of SUV39H1 correlates with a reduction in H3K9me3 levels Representative Western blots and bar graphs showing the signal quantifications. Averages of three samples ± SD. Expression levels were normalized to the expression in PD 38 cells.

The results of the differential gene expression analysis further showed that, when comparing PD 47 or PD 54 to PD 38 cultures, 2.3% and 2.7%, respectively, of the affected genes were involved in epigenetic regulation (Fig. 1C). Among these, *SUV39H1*, a histone methyltransferase that specifically methylates H3K9 and was previously associated with functions in aging or senescence (S4), was downregulated in senescent cells (PD 54), as confirmed by Western blot analysis (Fig. 3). This downregulation corresponded with significantly reduced H3K9 trimethylation and a trend towards increased H3K9 acetylation (Fig. 3). The loss of H3K9me3, a heterochromatin mark, suggests a global loss of heterochromatin in senescent cells. This may affect senescence through the induction of gene expression changes or genomic instability, and may thus play a causal role in the establishment of senescence.

### Reduced levels of SUV39H1 and H3K9me3 correlate with satellite expression

**Figure 4:**
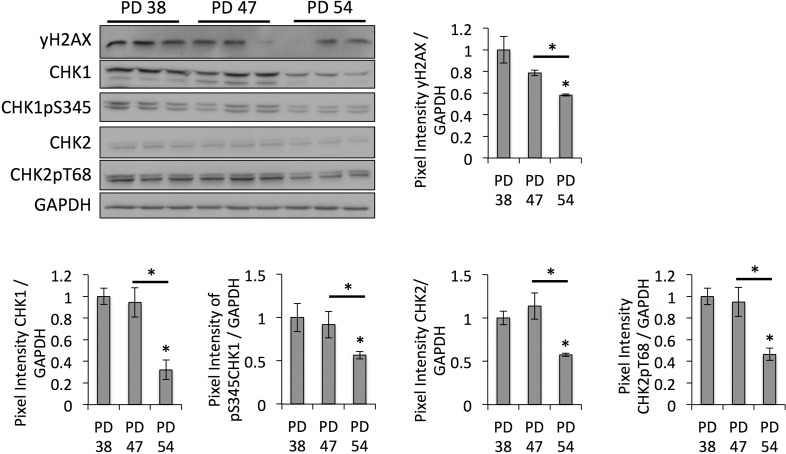
DNA damage checkpoint regulators are downregulated in senescent cells Representative Western blot images and bar graphs showing the quantification of the band intensities. Averages of three biological replicates ± SD.

Since SUV39H1-mediated H3K9 trimethylation is important for heterochromatin formation in pericentric satellite regions and, thus, for genomic stability [31, 32], we hypothesized that the downregulation of *SUV39H1* and the reduction of H3K9 trimethylation levels may contribute to the senescence-dependent loss of heterochromatin, resulting in genomic instability. In order to test this, we monitored the protein levels of DNA damage checkpoint regulators. This showed that CHK1 and CHK2 protein levels, as well as their phosphorylated forms, were lower in PD 54 cultures when compared to PD 38 and PD 47 cultures (Fig. 4). This is in line with the observed down-regulation of numerous transcripts involved in cell cycle regulation in general (S3).

**Figure 5:**
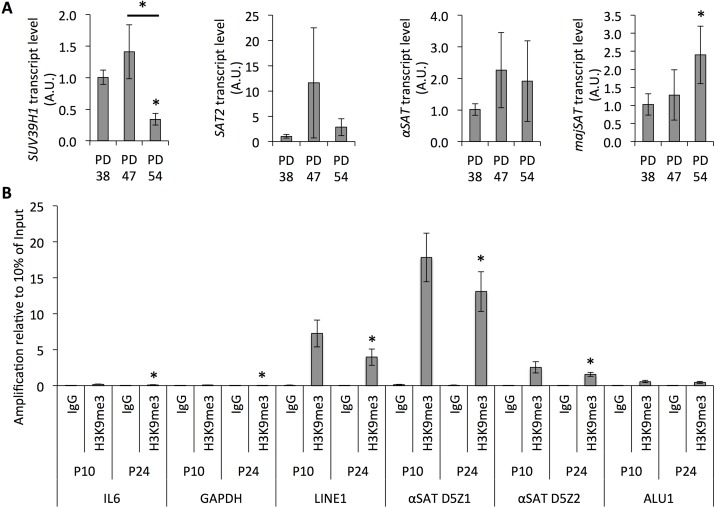
Senescence-dependent increase in satellite expression correlates with the loss of H3K9 trimethylation from those sequences (**A**) Transcript levels of *SUV39H1* and satellites normalized to *HPRT* and *YWHAZ* and to transcript levels in PD 38. (**B**) Bars represent averages from three biological replicates, error bars show the error progression of SDs.

Next, we examined whether the reduced SUV39H1 expression in senescent cells was associated with heterochromatin relaxation in pericentric satellite regions. The analysis of the transcript levels derived from different satellite regions showed a trend towards increased expression with the rising PD level of the cell cultures (Fig. 5A). This further correlated with the reduced amount of trimethylated H3K9 in satellite regions and in transposon sequences (Fig. 5B), as determined by ChIP. Therefore, the reduced SUV39H1 expression is associated with increased genomic instability through the loss of the H3K9me3 heterochromatin mark in regions of constitutive heterochromatin.

### Reduced SUV39H1 expression and H3K9me3 level during senescence may affect gene expression

**Figure 6:**
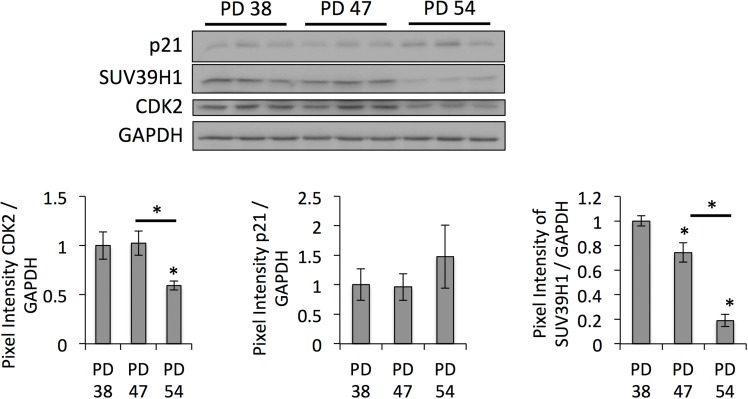
Downregulation of SUV39H1 affects gene expression profiles Western blot images and bar graphs showing the quantification of band intensities. Averages from three samples ± SD.

In addition to protecting the genome integrity, H3K9 trimethylation is also important for the transcriptional repression of protein-coding genes. For instance, RB recruits SUV39H1 to its target promoters to mediate transcriptional repression [33] and may therefore limit the expression of genes that are required for the active cell cycle. On the other hand, SUV39H1-mediated H3K9 trimethylation is involved in the silencing of the *p21* promoter [34]; thus, the loss of H3K9 trimethylation in the *p21* promoter results in *p21* expression associated with senescence. Therefore, we examined whether the altered H3K9me3 distribution patterns across the genome may contribute to senescence-dependent changes in gene expression. p21 protein levels were increased in senescent cells and correlated with the reduced expression levels of SUV39H1 (Fig. 6). Further, H3K9me3 was less abundant in the promoter region of *IL6* in senescent cells when compared to dividing cells (Fig. 5B), as determined by ChIP, suggesting that reduced SUV39H1 expression levels affect the gene expression profiles of senescent cells.

### SUV39H1 inhibition is more toxic for dividing than for senescent cultures

Since SUV39H1 downregulation contributes to increased genomic instability as well as senescence-associated gene expression, we hypothesized that SUV39H1 may play a causal role in the establishment of senescence. If so, the inhibition of SUV39H1 in dividing cells may induce senescence. In order to test this, we treated cells at different PD levels with chaetocin, a specific inhibitor of SUV39H1 [35].

**Figure 7:**
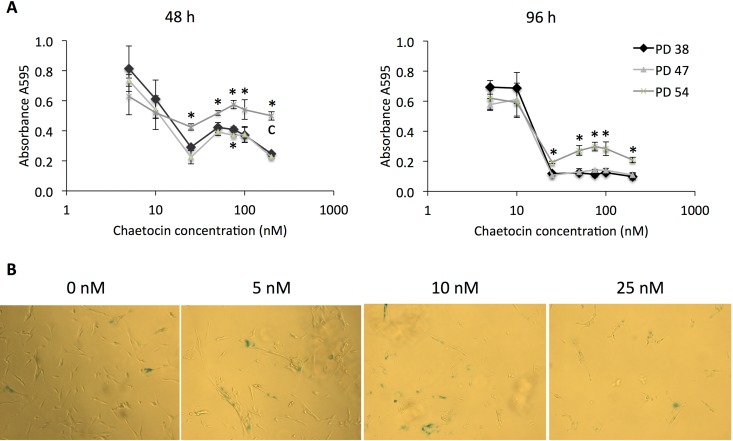
Chaetocin is more cytotoxic and induces senescence in low PD cultures (**A**) Cell viability after treatment with different concentrations (5 nM, 10 nM, 25 nM, 50 nM, 75 nM, 100 nM, or 200 nM) of chaetocin as determined by the MTT assay. Averages of measurements from three different cultures ± SD. (**B**) SA-β-GAL staining of PD 47 cultures treated with 0 nM, 5 nM, 10 nM, and 25 nM of chaetocin. Pictures were taken at 10× magnification.

First, we tested the cytotoxicity of increasing concentrations of chaetocin in the cells for all three PD levels, and found that cell cultures of all senescence states maintained a high level of viability when treated with 5 or 10 nM chaetocin and decreased rapidly at higher concentrations (Fig. 7A). Interestingly, the PD 54 cells, which express lower levels of SUV39H1 (Fig. 3), were more tolerant to higher concentrations of chaetocin, as seen by their viability at 48 h and 96 h following chaetocin treatment. In addition to a decrease in viability, increasing concentrations of chaetocin induced greater senescence in PD 47 cells (Fig. 7B).

### SUV39H1 overexpression in senescent cells induces cell division

**Figure 8:**
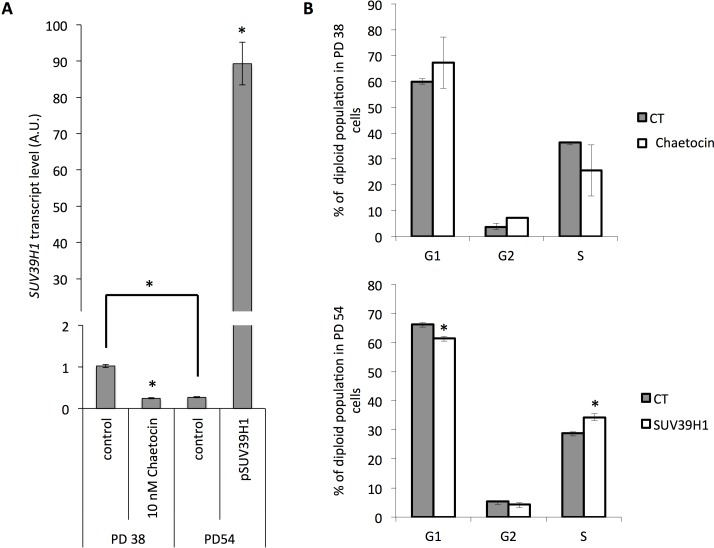
Modification of SUV39H1 expression levels affects cell cycle distribution (**A**) *SUV39H1* transcript levels normalized to PD 38 control. Averages of three samples ± error progression of SDs. (**B**) Cell cycle distribution. Averages of three samples ± SD.

Since treatment with increasing concentrations of chaetocin not only affected cell viability but also increasingly induced senescence in the exposed cells (Fig. 7B), we tested how the inhibition of chaetocin in PD 38 cells or the over-expression of *SUV39H1* in PD 54 cells affected cell division. For this, PD 38 cells were treated with DMSO or 10 nM chaetocin, while PD 54 cells were transfected with *pCMV6-SUV39H1*. Chaetocin treatment of PD 38 cells resulted in a reduction of *SUV39H1* transcript levels (Fig. 8A) and in a slight decrease in the number of cells in the S phase, which was not significant due to a high variation between the samples (Fig. 8B). On the other hand, the overexpression of *SUV39H1* in PD 54 cells resulted in increased *SUV39H1* transcript levels (Fig. 8A) and in a significant increase in the amount of cells in the S phase, with a corresponding significant decrease in the number of cells in the G1 phase (Fig. 8B).

## DISCUSSION

The cell cultures used herein were selected to show increasing PD levels and an increasing senescence ratio, along with reduced proliferation. The increasing senescence ratio was also associated with a higher number of cells with G2 DNA content. This is in line with previous reports which show that a large fraction of senescent cells exhibit polyploidy and arrest in the G2 phase of the cell cycle [26, 36].

The comparison of the gene expression profiles of three cultures with different senescence states (PD 38, PD 47, and PD 54) further supported the observations that PD 54 cultures contained significantly more senescent and less proliferating cells than the younger cultures; this was observed by the high number of genes involved in cell cycle regulation and aging or senescence affected by differential expression, thus supporting the validity of the model system.

In order to elucidate the underlying mechanisms involved, the gene expression profile of senescent cells was compared to that of young cells. The functional classification of genes that were differentially expressed between PD 47 and PD 38 as well as between PD 54 and PD 38 indicated that cell signalling and transcriptional and epigenetic regulation are affected by expression changes early on during the establishment of senescence (Fig. 1). Further, the regulatory network analysis that was performed using RENATO software connected the deregulation of 295 genes to transcriptional regulation by E2F1. E2F1, E2F2 and E2F3 are transcriptional activators, which are involved in the regulation of G1/S progression and DNA replication [28]. While all of the E2F transcription factors recognize the E2F recognition site in the promoter regions, their target specificity is thought to depend on their interaction with transcription co-activators or co-repressors, as well as on the presence of other binding motifs within the target genes [37-39]. While we did not observe any change in the expression levels of E2F1, *E2F2* was downregulated in senescent cells. As E2F1 and E2F2 both recognize the E2F recognition site used to screen for target genes, E2F2 may at least partially be involved in the regulation of the expression of the network associated with E2F1. Thus, the senescence-associated downregulation of E2F2 may play a role in the downregulation of genes involved in the G1/S progression and thus promote the G1 cell cycle arrest and the establishment of senescence. An involvement of E2F2 in the establishment senescence was previously shown in T cells, where E2F2 was shown to act as a transcriptional repressor to result in the inactivation of cell cycle genes and thus the promotion of the transition into the G0 phase of the cell cycle [40]. Further, senescence-associated heterochromatin formation was shown to be targeted to E2F target promoters by Rb [19].

The RENATO analysis also showed a significant enrichment of NF-YA targets among the differentially expressed genes when comparing PD 38 and PD 54. NF-YA also regulates the expression of genes involved in the G1/S transition, and has been previously shown to exhibit a decline in protein levels without a change in transcript level with increasing senescence in IMR90 cells [41]. In addition, E2F transcription factors have been shown to cooperate with other transcription factors, including NF-Y, to mediate the expression of cell cycle genes [42]. Thus, E2F2 and NF-Y may cooperate to mediate the G1 cell cycle arrest at the gene expression level.

Further analysis of the transcription factors that were deregulated during senescence and the correlation of their expression changes with those of their predicted target genes, implicated additional transcription factors that may be involved in the regulation of the senescence-associated gene expression profile. However, their TFBS were not enriched in the promoters of the differentially expressed genes when compared to all promoters. Thus, our findings and previous reports [40, 41, 43] support the important role of changes in transcriptional regulation in the establishment of senescence, in particular through the repression of cell cycle regulators.

In addition to changes in transcriptional regulation, the gene expression profiling data also indicated the deregulation of genes involved in the epigenetic regulation of senescent cells, such as *SUV39H1*, which was downregulated in senescent cells. This correlated with reduced global levels of H3K9 trimethylation (Fig. 3), site-specific reduction of H3K9me3 abundance in the promoter region of *IL6* and in regions of repetitive DNA sequences and corresponding transcription of satellite transcripts (Fig. 5), and with the increased expression of p21 (Fig. 6). This is in line with previous reports describing an important role of SUV39H1 in heterochromatin formation in pericentric satellite regions [31, 32, 44]. Additionally, such a senescence-dependent loss of constitutive heterochromatin has been previously described through the mapping of open chromatin regions by formaldehyde assisted isolation of regulatory elements in human diploid fibroblasts [45]. However, here we present the first evidence that the down-regulation of SUV39H1 in senescent human diploid fibroblasts contributes to heterochromatin relaxation in pericentric satellite regions and thereby contributes to genomic instability. Further, a function of SUV39H1 in the altered regulation of gene expression has also been described. SUV39H1 has been shown to silence S phase genes in terminally differentiating cells in cooperation with E2F/RB [46]. On the other hand, SUV39H1 also plays a role in the silencing of the *p21* promoter [34] and thus the loss of H3K9 trimethylation in the *p21* promoter results in *p21* expression associated with senescence. At first sight, these results may be contradictory, as some promoter regions are specifically targeted by SUV39H1 during senescence and in some promoter regions SUV39H1 is specifically lost. This may be due to the specific recruitment of SUV39H1 by transcription factors [46, 47], and may therefore depend on altered expression or activity of transcriptional programs. Additionally, the specific recruitment of SUV39H1 by transcription factors may contribute to the recruitment of the more limited pool of SUV39H1 away from the DNA regions that it is constitutively associated with. Nevertheless, this would need further investigation. Additionally, it would be important to analyze the roles of other histone H3 acetylation in cellular senescence, since recent reports show that stress-induced acetylation of H3 may regulate p21 transcription [48].

**Figure 9:**
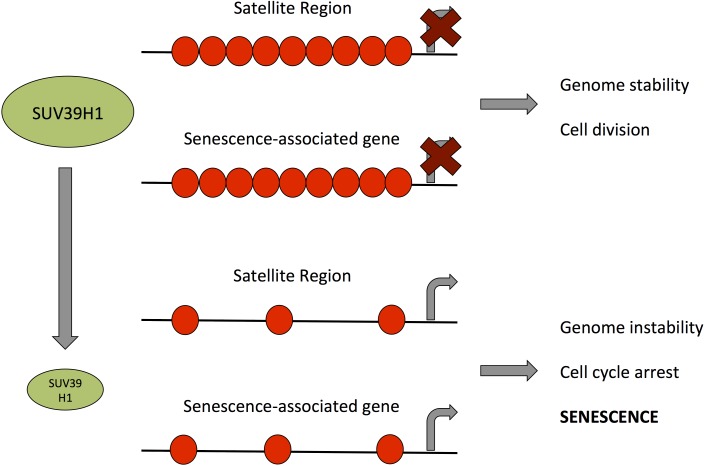
Model for the role of SUV39H1 down-regulation in the establishment of senescence Green ellipses represent the amount of SUV39H1 present in a cell, black lines represent DNA and red circles represent H3K9me3. Bent arrows indicate transcriptional activity.

The findings from previous reports [32, 49, 50] along with our observations that reduced SUV39H1 expression and reduced H3K9 trimethylation result in genomic instability and altered gene expression, which may both independently trigger senescence (Fig. 9), suggest that SUV39H1 may have a central function in the establishment of senescence. In line with this, PD 38 and PD 47 cells were significantly less tolerant to high concentrations (more than 25 nM) of chaetocin, a specific SUV39H1 inhibitor, when compared to PD 54 cells (Fig. 7). This may be due to the already reduced expression levels of SUV39H1 in PD 54 cells, suggesting that the cytotoxicity of chaetocin partially depends on the presence of its target SUV39H1. Further, the surviving cells in PD 47 cultures treated with increasing concentrations of chaetocin became senescent (Fig. 7B), suggesting that the inhibition of SUV39H1 was sufficient to induce senescence in low PD cells. Additionally, the inhibition of SUV39H1 in PD 38 cells resulted in a decrease in the amount of cells in the S phase, whereas the overexpression of *SUV39H1* in PD 54 cells induced cell division (Fig. 8). These results support the important role of SUV39H1 in the regulation of the transition between cell division and senescence. In addition to our results, previous reports have described several functions of SUV39H1 that underline its importance in cell division such as its role in chromatin organization at centromeres during cell division [51], its role in the organization of the nuclear architecture [52], the inhibition of cellular differentiation programs in transgenic Suv39h1 over-expressing mice [49], and the fact that suv39h1/2 knockout mice suffered from reduced viability, genomic instability, and susceptibility to cancer [32]. While a role for SUV39H1 in the establishment of heterochromatin foci has been described [50], herein we suggest that, during the establishment of replicative senescence, the downregulation of SUV39H1 might provide a switch that alters the chromatin structure from a conformation that promotes cell division to one that promotes cell cycle arrest (Fig. 9). Support for such a theory can be found in a recent study showing that the relaxation of satellite heterochromatin is an early event in the establishment of senescence [53].

However, further studies are needed to elucidate how this downregulation of SUV39H1 is initiated and whether it is a gradual process or an acute switch from one state to the other. Moreover, more studies are needed to dissect the roles of SUV39H1 in aging and replicative senescence. A recent study by Liu et al. (2013) reported that Zmpste24(-/-)/Suv39h1(-/-) mice had a longer maximum lifespan than Zmpste24(-/-) mice – 29 weeks as compared to 40 weeks [54]. Interestingly, Zmpste24 deficiency was shown to be associated with accumulation of unrepaired DNA damage (similar to the one observed in senescent cells) and compromised recruitment of checkpoint and DNA repair proteins to damage sites [55]. Therefore, since Suv39h1 downregulation was also shown to be beneficial for heterochromatin DNA repair [56], it is plausible that Suv39h1 knock-out in Zmpste24(-/-) mice results in slightly more efficient DNA repair and extended lifespan. Notwithstanding, these results may not directly relate to the function of Suv39h1 in replicative senescence. Indeed, it is certainly plausible that the SUV39H1 down-regulation may occur due to the accumulation of unrepaired DNA damage and may initially help the cells repair some of the damage, however, this may be followed by increased genomic instability due to de-heterochromatinization of repetitive DNA sequence elements and thereby contribute to the establishment of senescence.

In summary, the present study has shown that replicative senescence in WI-38 cells is associated with major changes to the gene expression profile. Some of these changes may be caused by altered transcriptional regulation. In addition, we describe a role for the senescence-associated downregulation of SUV39H1 in the loss of heterochromatin in satellite regions, as well as in the promoters of protein coding genes. These changes in chromatin structure may promote the induction of senescence and, in line with this the overexpression of *SUV39H1* in senescent cells, induces cell division, whereas the inhibition of SUV39H1 in dividing cells inhibits cell division. Therefore, we suggest that SUV39H1 functions as a switch by maintaining a chromatin conformation that is favourable to cell division as long as it is expressed.

## METHODS

### 

#### Cell culture

Human foetal lung fibroblasts (WI-38, ATCC, CCL-75TM) were maintained in HyClone minimum essential medium (MEM) Alpha Modification (Thermo Scientific, Waltham, MA, USA) containing 10% (v/v) foetal bovine serum (FBS) (Gibco/Invitrogen, Burlington, ON, Canada) in a humidified Forma Steri-Cycle CO_2_ Incubator (Thermo Scientific) at 37°C and 6% CO_2_. The population doubling (PD) number of a given passage was determined by summing up the ΔPD = log_2_(n_f_/n_i_) for each subculture, where n_f_ is the final number of cells in a culture and n_i_ is the number of cells initially inoculated.

### Senescence-associated β-galactosidase (SA-β-GAL) assay

#### Flow cytometry-based assay

A fluorescent SA-β-GAL staining assay was performed according to a previous protocol involving the incubation of cells with 5-dodecanylaminofluorescein di-β-D-galactopyranoside (C_12_FDG, Sigma-Aldrich Canada Ltd., Oakville, ON, Canada) at standard culture conditions for 1.5 h [57]. The cells were analyzed on the BD FACS Canto II (BD Biosciences, Franklin Lakes, NJ). Three samples of each PD level were analyzed by detecting 10,000 events per sample.

#### Microscopy-based assay

For the microscopy-based analysis of SA-β-GAL activity, cells were fixed and stained using the SA-β-GAL Staining Kit (Cell Signaling, Bedford, MA). Images were taken using a Zeiss Observer Z1 epifluorescence microscope with AxioVision Rel 4.8 software.

Determination of cell cycle distribution. Cells were suspended in 1mL PBS and fixed in 63% ethanol for 48 h at -20°C. DNA staining was performed in staining solution (0.1% Triton-X-100, 20 µg/ml propidium iodide, and 20 µg/ml Ribonuclease A in PBS) for 15 min at 37°C. Samples were analyzed on a BD FACS Canto II (BD Biosciences) and 10,000 events were detected per sample.

#### Gene expression profiling

Total RNA was extracted from three samples per PD level using TRIzol^®^ Reagent (Invitrogen, San Diego, CA). Gene expression profiles were determined using Illumina^®^ HumanHT12-v4 Gene Expression BeadChips according to the manufacturer's protocol. Differential expression analyses were performed using the Illumina^®^ GenomeStudio software using an Illumina-custom model with an FDR of 0.05 applied; PD 38 was used as a reference. Only genes for which the differential expression analysis was significant based on *p* <0.05 and the log2-fold change was smaller than -0.4 or larger than 0.4, were considered for further analysis.

### Bioinformatics analyses

#### Sample clustering

Sample clustering was performed using the Illumina^®^ GenomeStudio software.

#### Functional classification

Functional classification of genes was performed using FunNet Transcriptional Networks Analysis (www.funnet.info), g:Profiler [58, 59], RENATO [60], and DAVID Bioinformatics Resources 6.7 [61] software and compared with information on the genes available from the Genecards database (http://www.genecards.org; [62].

#### Transcription factor analysis

Promoter sequences of 1,000 bases upstream of the transcription start sites of the RefSeq genes from the RefSeq release 37 (November 2009, GRCh37/hg19, http://hgdownload.soe.ucsc.edu/goldenPath/hg19/bigZips/upstream1000.fa.gz; [63]) were searched for transcription factor binding sites (TFBS) of transcription factors affected by differential expression (retrieved from Genecards www.genecards.org; [62] or Bio-Base https://portal.biobase-international.com/) using the PatMatch v1.2 program (ftp://ftp.arabidopsis.org/home/tair/Software/Patmatch/patmatch_1.2.tar.gz; [64] downloaded from the TAIR website [65] without allowing mismatches.

Only promoters which contained a higher or lower than the mean ± standard deviation (SD) number of occurrences of a specific TFBS when compared to all the promoters of all RefSeq genes were considered for further analysis. Additionally, a correlation analysis was performed to determine genes for which the change in gene expression correlated with the change in transcription factor expression.

#### Quantitative real-time PCR (qRT-PCR)

qRT-PCR reactions were set up using the SsoFast^™^ EvaGreen^®^ Supermix (BioRad) and primers specific for target sequences of interest (S5). The reactions were analyzed on a C1000^™^ Thermo Cycler equipped with a CFX96^™^ Real-Time System (BioRad) according to the SSoFast^™^ guidelines with annealing temperatures as specified for the specific primer pairs (S5).

Each experiment included three biological replicates for each PD level and two technical replicates per sample. Normalization using three housekeeping genes - *HPRT1*, *RPL13A*, and *YWHAZ* and the analysis of the qRT-PCR Ct results were carried out using qbase^PLUS^ [66].

#### Western immunoblotting

For protein isolation, cells were sonicated in 100 µl cold 1% sodium dodecyl sulphate (SDS) containing protease inhibitor (Roche, Basel, Switzerland). Western blots were performed as previously described [67], and the membranes were incubated with primary antibodies overnight at 4°C (S6). Chemiluminescence was detected using a FluorChem HD2 camera with the FluorChem software (Cell Biosciences, Santa Clara, CA). Band intensities were quantified using NIH Image J64 software and normalized relative to GAPDH.

#### MTT cytotoxicity assay

For the determination of cell viability, approximately 5,000 cells were plated per well in a 96-well plate. Various concentrations of chaetocin, as indicated, or DMSO as a control, were added to the cultures 24 h after plating. The cell viability was determined using the Cell Proliferation Kit I (Roche) 48 h or 96 h after the addition of chaetocin.

#### SUV39H1 inhibition and overexpression

In order to inhibit SUV39H1, cells were treated with a 10 nM final concentration of chaetocin, which is a specific inhibitor of SUV39H1 [35]. For the overexpression of *SUV39H1*, cells were transfected with the pCMV6-SUV39H1 expression plasmid (Origene, Rockville, MD) using Lipofectamine^™^ 2000 (Invitrogen).

#### Chromatin immunoprecipitation (ChIP)

ChIP was performed as described previously [68]. Immuno-precipitation was performed by using antibodies targeted to rabbit anti-H3K9me3 (Abcam) or mouse anti-SUV39H1 (Abcam), or rabbit or mouse IgG as a negative control. Following immunoprecipitation, DNA fragments were isolated using 10% Chelex^®^100 and purified using a QIAquick^®^ PCR purification kit.

ChIP-pRT-PCR was performed the same way as described above using the primers listed in S7.

#### Statistical analysis

All experiments included three biological replications and statistically significant differences were determined by pairwise, two-tailed Student's *t*-test (*p* <0.05).

## SUPPLEMENTARY MATERIAL






