# Supplementary material for: A role for SUV39H1-mediated H3K9 trimethylation in the control of genome stability and senescence in WI38 human diploid lung fibroblasts
Source: Aging (Albany NY). 2014 Jul 21;6(7):545–63. doi: 10.18632/aging.100678 (PMC4153622; doi:10.18632/aging.100678)
Supplement: Supplementary file 3 [file aging-06-545-s003.pdf]

SUPPLEMENTAL MATERIAL

Please browse the full text version of this manuscript to see Supplemental Tables 1 and 2.

S3. Changes in the expression of cell cycle regulators.
Numbers represent up- or down-regulated genes when compared to the total number of genes with expression changes in the specified subcategory. Arrows next to category title indicate whether the numbers reflect fractions of up- or down-regulated genes.

Sub-Category	PD 47	PD 54
**Cell cycle progression**		
**Inhibitors ↑**	0	4 / 10
**Activators ↓**	0	6 / 10
**G1 progression**		
**Inhibitors ↑**	0	3 / 11
**Activators ↓**	0	13 / 15
**S phase progression ↓**	0	12 / 12
**DNA replication ↓**	0	46 / 47
**G2 progression ↓**	0	9 / 11
**Mitosis ↓**	0	81 / 87

S4. Senescence-associated gene expression.
Entries of up-regulated or down-regulated genes with age are based on microarray signatures from the Human Ageing Genomic Resources [47]. Other gene information is based on Genecards (http://www.genecards.org/; [32]. Numbers indicate log2-fold changes in transcript levels when comparing either PD 47 or PD 54 to PD 38 cells.

Gene	Involvement in ageing	PD 47	PD 54
**ANXA3**	Up-regulated with age		-0.46
**C5ORF13**	Down-regulated with age		-0.62
**CDKN1B**	Up-regulated with age		-0.57
**COL1A1**	Down-regulated with age		-0.46
**COL3A1**	Down-regulated with age		–1.05
**DKC1**	May regulate telomerase activity		–0.45
**GBP2**	Up-regulated with age		–0.75
**HIST1H1C**	Up-regulated with age		0.58
**ID2**	Down-regulated with senescence	0.60	0.79
**MNT**	May induce senescence		0.46
**PRKDC**	Telomere maintenance		–0.41
**RAD54L**	Telomere maintenance		–0.65
**SUV39H1**	H3K9methylation dependent induction of senescence		–0.75
**TFRC**	Down-regulated with age		–0.51
**TIMP3**	May be involved in senescence		0.95
**TMED10**	Up-regulated with age		0.58
**TXNIP**	Up-regulated with age		1.52

S5. Primers for qRT-PCR.

Gene	Fwd/ Rev	Sequence	Annealing Temp	Reference
HPRT1	Fwd<br>Rev	5’-TGACACTGGCAAAACAATGCA-3’<br>5’-GGTCCTTTTCACCAGCAAGCT-3’	59.5°C	[37]
YWHAZ	Fwd<br>Rev	5’-ACTTTTGGTACATTGTGGCTTCAA-3’<br>5’-CCGCCAGGACAAACCAGTAT-3’	59.5°C	[37]
RPL13A	Fwd<br>Rev	5’-CCTGGAGGAGAAGAGGAAAGAGA-3’<br>5’-TTGAGGACCTCTGTGTATTTGTCAA-3’	59.5°C	[37]
COL3A1	Fwd<br>Rev	5’-CGATGAGATTATGACTTC-3’<br>5’-ATTACAGAATACCTTGATAG-3’	53.0°C	
UBE2C	Fwd<br>Rev	5’-ACATATGCCTGGACATCCTGA-3’<br>5’-GGTTCTCCTAGAAGGCTCTGG-3’	59.5°C	[38]
POT1	Fwd<br>Rev	5’-<br>GGGCAAAGCAGAAGTGGACGGAGCATC-<br>3’<br>5’-<br>ATTGACAGATAACATCTGAATGCTGATTG<br>GCTGTC -3’	59.5°C	[39]
FOXM1	Fwd<br>Rev	5’-ACTTTAAGCACATTGCCAAGC-3’<br>5’-CGTGCAGGGAAAGGTTGT-3’	55.9°C	[40]
E2F2	Fwd<br>Rev	5’-CCAAGAATTACATCAGAGAA-3’<br>5’-GCTTACATTCCAGACTTC-3’	55.9°C	
SUV39H1	Fwd<br>Rev	5’-CTACTATGGCAACATCTC-3’<br>5’-GTCAAGGTTGTCTATGAA-3’	55.9°C	
SAT2	Fwd<br>Rev	5’-CATCGAATGGAAATGAAAGGAGTC-3’<br>5’-ACCATTGGATGATTGCAGTCAA-3’	59.5°C	[41]
majSAT	Fwd<br>Rev	5’-GACGACTTGAAAAATGACGAAATC-3’<br>5’-CATATTCCAGGTCCTTCAGTGTGC-3’	55.9°C	[41]
alphaSAT	Fwd<br>Rev	5’-CTGCACTACCTGAAGAGGAC-3’<br>5’-GATGGTTCAACACTCTTACA-3’	55.9°C	[41]

S6. Antibodies for Western blots.

Target	Supplier, Cat No	Dilution
**Mouse anti-SUV39H1**	Abcam, ab12405	1:500 in 5% milk (PBST)
**Rabbit anti-H3K9me3**	Abcam, ab8898	1:500 in 5% milk (PBST)
**Rabbit anti-H3K9ac**	Abcam, ab10812	1:500 in 5% milk (PBST)
**Rabbit anti-H3**	Cell Signaling, 9715	1:1,000 in 5% milk (PBST)
**Rabbit anti-CHK2**	Abcam, ab8108	1:250 in 5% milk (PBST)
**Rabbit anti-pT68CHK2**	Abcam, ab3501	1:500 in 5% milk (PBST)
**Mouse anti-CHK1**	Cell Signaling, 2360	1:1,000 in 5% BSA (PBST)
**Rabbit anti-pS345CHK1**	Cell Signaling, 2341	1:1,000 in 5% milk (PBST)
**Rabbit anti-p21**	Abcam, ab7960	1:1,000 in 5% milk (PBST)
**Mouse anti-GAPDH**	Santa Cruz, sc47724	1:1,000 in 5% milk (PBST)
**Mouse anti-ACTIN**	Abcam, ab3280	1:1,000 in 5% BSA (PBST)
**Secondary Antibodies**		
**Donkey anti-Rabbit**	Santa Cruz, sc2313	1:10,000 in 5% milk (PBST)
**Goat anti-Mouse**	Santa Cruz, sc2005	1:5,000 in 5% milk (PBST)

S7. Primers for ChIP-qRT-PCR.

Target	Fwd/ Rev	Sequence	Annealing Temp	Reference
IL6	Fwd<br>Rev	5'-CTTCGTGCATGACTTCAGCTTT-3'<br>5'-CGTCCTTTAGCATCGCAAGAC-3'	62.5°C	
GAPDH	Fwd<br>Rev	5'-TACTAGCGGTTTTACGGGCG-3'<br>5'-TCGAACAGGAGGAGCAGAGAGCGA-3'	62.5°C	[44]
ALU1	Fwd<br>Rev	5'-ACGAGGTCAGGAGATCGAGA-3'<br>5'-CTCAGCCTCCCAAGTAGCTG-3'	51°C	[45]
LINE1	Fwd<br>Rev	5'-CAGAATCTCTGGGACGCATT-3'<br>5'-ATTGTGATGTTCGGGTGTCA-3'	64.1°C	[45]
αSAT D5Z1	Fwd<br>Rev	5'-TAGACAGAAATATTCTCACAATCGT-3'<br>5'-GCCCTCAAAGCGCTCCAAG-3'	62.5°C	[45]
αSAT D5Z2	Fwd<br>Rev	5'-TTTTTGTGCAATTGGCAAATGGAG-3'<br>5'-AGACTGTTTCCTCACTGCTCT-3'	62.5°C	[45]
